# P-1185. Healthcare Resource Utilization (HCRU) of Critical Care Patients by Rank of Initiation from the Study of Prescribing patterns and Effectiveness of Ceftolozane/Tazobactam: Real-world Analysis (SPECTRA)

**DOI:** 10.1093/ofid/ofaf695.1378

**Published:** 2026-01-11

**Authors:** Emre Yucel, Alex Soriano, Florian Thalhammer, Stefan Kluge, Pierluigi Viale, Mike Allen, Brune Akrich, Jessica Levy, Huina Yang, Sundeep Kaul

**Affiliations:** Merck & Co., Inc., North Wales, PA; Hospital Clínic de Barcelona, Barcelona, Catalonia, Spain; Medizinische Universität Wien, Vienna, Wien, Austria; Department of Intensive Care, University Medical Center Hamburg-Eppendorf, Hamburg, Hamburg, Germany; Infectious Diseases Unit, Department of Medical and Surgical Sciences, Policlinico Sant'Orsola Malpighi, University of Bologna, Bologna, Italy, Bologna, Emilia-Romagna, Italy; MSD, London, England, United Kingdom; Merck Research Labs, MSD, Puteaux, Ile-de-France, France; Merck & Co., Ltd, Rahway, New Jersey; Tan Tock Seng Hospital, Singapore, Not Applicable, Singapore; Harefield hospital, london, England, United Kingdom

## Abstract

**Background:**

A sub-analysis of SPECTRA (Study of Prescribing patterns and Effectiveness of Ceftolozane/Tazobactam Real-world Analysis) aimed to analyze healthcare resource utilization among 298 critical care patients stratified by the rank of chemotherapy or targeted therapy (C/T) initiation, focusing on 30-day all-cause readmissions, infection-related readmissions, prolonged hospital stays, and median length of hospital stays post-C/T initiation (n=298).

**Figure:**
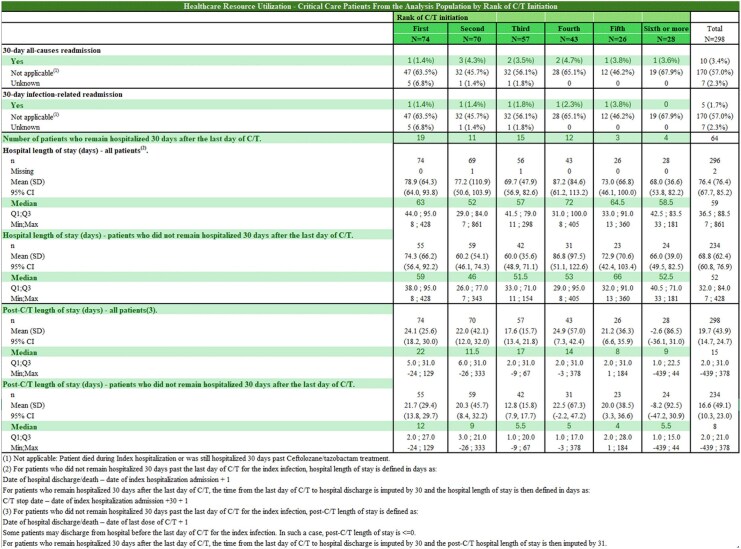

**Methods:**

SPECTRA was a multicenter, observational study in patients (≥18 years of age) treated with ≥48 h of C/T in a hospital setting. For HCRU, data were categorized by the rank of C/T initiation: First Rank (N=74), Second Rank (N=70), Third Rank (N=57), Fourth Rank (N=43), Fifth Rank (N=26), and Sixth or more Rank (N=28). The study examined 30-day all-cause readmission rates, 30-day infection-related readmission rates, the number of patients remaining hospitalized 30 days post-C/T initiation, and median hospital length of stay overall and post-C/T initiation for patients discharged within 30 days.

**Results:**

30-day All-Cause Readmission Rate was 3.4% (variations:1.4% to 4.7% across ranks). 30-day infection-related readmission rate averaged 1.7% overall, (variations: 0% to 3.8%). Patients remaining hospitalized 30 Days after C/T initiation (n=64; 21.5%) were predominantly in the first four ranks. Median hospital length of stay (LOS) was 59.0 days (52.0-72.0). Median Post-C/T LOS for patients discharged within 30 Days was 8.0 days overall, with durations ranging from 4.0 to 12.0 days.

**Conclusion:**

In conclusion, the examination of HCRU among critical care patients in the SPECTRA study reveals variations in readmission rates and hospital lengths of stay by the rank of C/T initiation. Further research is warranted to explore factors influencing these variations in HCRU, by novel antibiotics such as C/T. Future research may benefit antimicrobial stewardship and resource management in critical care environments.

**Disclosures:**

Emre Yucel, PhD, Merck & Co., Ltd: Stocks/Bonds (Public Company) Jessica Levy, n/a, Merck & Co., Ltd: Stocks/Bonds (Public Company)

